# Effects of Inhibitors on the Transcriptional Profiling of *Gluconobater oxydans* NL71 Genes after Biooxidation of Xylose into Xylonate

**DOI:** 10.3389/fmicb.2017.00716

**Published:** 2017-04-25

**Authors:** Yuanyuan Miao, Yi Shen, Yong Xu

**Affiliations:** ^1^Department of Bioengineering, College of Chemical Engineering, Nanjing Forestry UniversityNanjing, China; ^2^Jiangsu Province Key Laboratory of Green Biomass-based Fuels and Chemicals, Nanjing Forestry UniversityNanjing, China; ^3^Jiangsu Co-Innovation Center of Efficient Processing and Utilization of Forest Resources, Nanjing Forestry UniversityNanjing, China

**Keywords:** *Gluconobacter oxydans*, xylose, xylonate, lignocellulosic inhibitor, responsible gene, transcriptome sequencing

## Abstract

D-Xylonic acid belongs to the top 30 biomass-based platform chemicals and represents a promising application of xylose. Until today, *Gluconobacter oxydans* NL71 is the most efficient microbe capable of fermenting xylose into xylonate. However, its growth is seriously inhibited when concentrated lignocellulosic hydrolysates are used as substrates due to the presence of various degraded compounds formed during biomass pretreatment. Three critical lignocellulosic inhibitors were thereby identified, i.e., formic acid, furfural, and 4-hydroxybenzaldehyde. As microbe fermentation is mostly regulated at the genome level, four groups of cell transcriptomes were obtained for a comparative investigation by RNA sequencing of a control sample with samples treated separately with the above-mentioned inhibitors. The digital gene expression profiles screened 572, 714 genes, and 408 DEGs was obtained by the comparisons among four transcriptomes. A number of genes related to the different functional groups showed characteristic expression patterns induced by three inhibitors, in which 19 genes were further tested and confirmed by qRT-PCR. We extrapolated many differentially expressed genes that could explain the cellular responses to the inhibitory effects. We provide results that enable the scientific community to better define the molecular processes involved in the microbes' responses to lignocellulosic inhibitors during the cellular biooxidation of xylose into xylonic acid.

## Introduction

The biorefinery of lignocellulose and related bioproducts are currently of major concern. In particular, D-xylose presents an attractive opportunity for the production of biochemicals and biofuels as it is the second most abundant carbohydrate among the different components forming the lignocellulose (Akinterinwa and Cirino, 2009; Vleet and Jeffries, 2009; Nair and Zhao, 2010). Interestingly, D-xylonic acid belongs to the top 30 biobased platform chemicals. It is also found in a wide range of products such as concrete additive, oil well cement redarter, glass and melt conditioner, water stabilizer, and also in some pharmaceutical materials (Markham, 1990; Millner et al., 1994; Chun et al., 2003, 2006; Tomoda et al., 2004; Pujos and Jijakli, 2006). Furthermore, Niu et al. reported that D-xylonic acid could be used to produce 1,2,4-butanetriol, which is a valuable precursor for the strategic energetic material named 1,2,4-butanetriol trinitrate (Niu et al., 2003). Recently, Japanese and Chinese companies applied D-xylonic acid as a blending ingredient used for the cooling of the spinning during the production of woven cotton (Liu et al., 2012; Xu et al., 2012).

The basic conjugate D-xylonate can be produced at high yield from D-xylose by native bacteria and genetically modified microbes from the *Gluconobacter oxydans* strain (Buchert, 1990). The gram-negative acetic acid bacterium *G. oxydans* is a valuable biocatalyst used in industry because it can regioselectively oxidize sugars and sugar alcohols (Gupta et al., 2001). We previously reported the selection and isolation of the *G. oxydans* NL71 strain obtained from yearly consecutive cultures of crude lignocellulosic hydrolysates obtained from diluted sulfuric-acid pretreated agricultural stovers. The highest performance of D-xylonate bioproduction was so far observed with the *G. oxydans* NL71 strain that could produce 586.3 g/L of xylonic acid starting from 600 g/L of xylose, representing a yield of 95.1% and a volumetric productivity of 4.69 g/L/h (Zhou et al., 2015).

Although lignocellulosic biomass provided an abundant and economic source of D-xylose, the growth of *G. oxydans* during the xylonate bioproduction was seriously inhibited at a high concentration of D-xylose (Buchert et al., 1988; Buchert and Niemelä, 1991). The reason was rationally ascribed to the fact that the degraded chemicals formed during the lignocellulose pretreatment and hydrolysis stem from a fixed procedure of the lignocellulosic biorefinery system. Carbohydrate-degraded products (furfural, 5-hydroxymethylfurfural, formic acid, and laevulinic acid) are known to inhibit the growth of microorganisms. Moreover, lignin-degraded products, such as vanillic acid, vanillin, syringaldehyde, syringic acid, 1,4-dihydroxybenzene, and 4-hydroxybenzoic acid, are suspected to be even more toxic to the bacteria due to a synergistic or cumulative effect although they are present only in small amounts in the hydrolyzates. Detoxification by diethylether extraction, active carbon adsorption, and resin exclusion improves the bioconversion efficiency but involves an additional cost (Palmqvist and Hahn-Hägerdal, 2000a,b; Jönsson et al., 2013). Therefore, the approach of increasing the microbe's tolerance by understanding the key genes reacting with the inhibitors was attractive.

We previously reported that the critical inhibitory effects of the three typical degraded compounds, namely formate, furfural, and 4-hydroxybenzaldehyde (PHB), on the bioproduction of D-xylonate. Understanding the transcriptome was essential for interpreting the functional elements of the genome, so that we could reveal the gene roles during the inhibitory responses (Tadra-Sfeir et al., 2015; Wang et al., 2017). The transcriptome of *G. oxydans* was therefore analyzed using RNA-sequencing techniques in the case of the genome-wide transcriptional responses to oxygen limitation and of the glucose catabolism via a partially cyclic pentose phosphate pathway (Hanke et al., 2012, 2013). However, no studies have been published on the analysis of the transcriptome of *G. oxydans* by RNA-sequencing in the presence of typical inhibitors during the production of D-xylonate.

The objective of this study was to use high-throughput RNA-sequencing to generate the respective comprehensive transcriptome profiles of *G. oxydans* NL71 in culture media containing separately the three lignocellulosic inhibitors. The comparison of the various transcriptome profiles would reveal the genes responsible for inhibition and help to understand the molecular mechanisms associated to the bioconversion of D-xylose into D-xylonate by *G. oxydans* NL71.

## Materials and methods

### Bacterial strains, culture conditions, and collection of the strains

Colonies of *G. oxydans* NL71, isolated from the strain ATCC 621 and stored at the Nanjing Forestry University of China (Miao et al., 2015), were maintained on sorbitol agar plates (sorbitol 50 g/L, yeast extract 5 g/L, agar 15 g/L) at 4°C. Precultures of the strain were cultivated in simple medium (50 mL) containing sorbitol (100 g/L) and yeast extract (10 g/L) in Erlenmeyer shake flasks (250 mL) for 24–36 h at 30°C and 220 rpm. The initial optical density at 600 nm (OD_600_) was ca. 1. For the determination of the inhibitor's influence, cells were cultivated in 50 mL of the same cell-biocatalysis medium containing D-xylose (50 g/L), yeast extract (5.0 g/L), magnesium sulfate (MgSO_4_, 0.5 g/L), potassium diphosphate (KH_2_PO_4_, 1.0 g/L), potassium phosphate (K_2_HPO_4_, 2.0 g/L), and ammonium sulfate [(NH_4_)_2_SO_4_, 5.0 g/L; Jiang et al., 2016]. Before the whole-cell catalysis, formic acid, furfural, and 4-hydroxybenzaldehyde were separately added to the cell-biocatalysis medium. The control cells and the cells separately treated with the three different inhibitors were carefully collected. In order to isolate the RNA, the samples were placed into a clean microtube free of RNase and immediately frozen in liquid nitrogen or at −80°C for storage.

### RNA isolation, cDNA library construction, and sequencing

All the RNAs were extracted from the bacteria by using the TRIzol® Reagent (name of the supplier, country). The quality of the isolated RNAs was analyzed on 1% x/x agarose gels and analyzed by UV-vis spectroscopy (NanoPhotometer®, Implen Inc., USA). The RNA concentration was determined by the Qubit® RNA Assay Kit with the Qubit® 2.0 Fluorometer (Life Technologies Corp., USA) and the RNA integrity was assessed using the RNA Nano 6000 Assay (supplier) with the Bioanalyzer 2100 system (Agilent Technologies Inc., USA).

The RNA quality assessment was performed on a total amount of 3 μg of extracted RNA per sample. We constructed the library using four samples, which presented a RNA integrity number (RIN) value above 8. We used the NEBNext® Ultra™ RNA Library Prep Kit for Illumina® (New England BioLab's Inc., USA) to generate the sequencing libraries. Briefly, mRNA was purified from the total RNA by the bacterial specific Ribo-Zero™ Magnetic Kit (name of the supplier, country). Fragmentation was undertaken by using divalent cations at an elevated temperature in the NEBNext® First Strand Synthesis Reaction Buffer (5X, supplier, country). A random hexamer primer and M-MμLV Reverse Transcriptase (RNase H, supplier, country) was used to synthesize the first strand cDNA. Subsequently, DNA Polymerase I (supplier, country) and RNase H was used to synthesize the second strand cDNA. After adenylation of the 3′ ends of the DNA fragments, the ligation to the fragments of the NEBNext Adaptors presenting a hairpin loop structure was performed prior to hybridization. We used the AMPure XP system (Beckman Coulter, USA) to purify the library fragments in order toto be able to select cDNA fragments exhibiting a length of 150–200 bp. Then, excision of the uracil bases of the size-selected adaptor-ligated cDNA was catalyzed by adding the USER Enzyme (3 μl, concentration, New England BioLab's Inc., USA) and incubating at 37°C for 15 min followed by heating at 95°C for 5 min before the polymerase chain reaction (PCR). At last, the PCR products were purified (AMPure XP system, supplier, country) and the library quality was determined with the Agilent Bioananlyzer 2100 system (Agilent Technologies Inc., USA).

The clustering of the index-coded samples was performed on a cBot Cluster Generation System using the TruSeq PE Cluster Kit v3-cBot-HS (Illumina®, supplier, country) and by following the manufacturer's instructions. After the generation of the cluster, the library fragments were sequenced with the Illumina® Hiseq 2000 platform and the list of the 100 bp paired-end reads were generated.

### Quality control of the paired-end reads

We used the Illumina® sequencing platform (GAΠ) to sequence the cDNA library. The FASTQ-formatted raw data (raw reads) were firstly processed through inner perl scripts to remove the reads with an adapter or poly-N sequences and the ones of low quality, in order to obtain the clean data (clean reads). In parallel, the parameters of Q20 (the proportion of read bases whose error was <1%) and Q30 (the proportion of read bases whose error was <0.1%), and the GC contents of the clean data were calculated. All the following analyses were based on the high quality clean data.

### Quantification and differential expression analysis of transcripts

We could quantify the gene expression level by the reads count because the number of the RNA-seq reads obtained from a transcript reflected its abundance. The commonly used Cuffdiff (Trapnell et al., 2010) and DEGSeq (Anders and Huber, 2010) methods were used to identify the differentially expressed genes between the different groups.

In this study, we used the HTSeq v0.6.1 tool (http://www-huber.embl.de/users/anders/HTSeq) to count the numbers of reads mapped against each gene. The length of the gene and reads-count mapped against the same gene was used to calculate the reads per kilobase of exon per million mapped reads (RPKM) for each gene. The value of the RPKM considered the effects of the sequencing depth and gene length for the reads count at the same time and represented the most common estimation of the gene expression levels (Mortazavi et al., 2008; Roberts et al., 2011).

Alternative splicing events (AS events) were classified into 12 basic types by the software Asprofile v1.0 (http://ccb.jhu.edu/software/ASprofile/). The number of AS events in each sample was separately estimated (Sammeth et al., 2008).

Before the differential gene expression analysis, the edgeR program package was used to adjust the read counts by one scaling normalized factor for each sequenced library. We used the DEGSeq R package v1.12.0 (supplier, country or URL link) to analyze the differential expression of two conditions (Wang et al., 2010). The Benjamini and Hochberg method (Benjamini and Hochberg, 1995) was used to adjust the *P*-values and set the corrected *P*-value to 0.005. Additionally, the log_2_ (Fold-change) was set to 1 as the threshold value for significant differential expressions.

### GO and KEGG enrichment analysis

The GOseq R package (supplier, wountry or URL link) was used to analyze the gene ontology (GO) enrichment of the differentially expressed genes (Young et al., 2010), as the gene length bias could be corrected within the package. The GO terms presenting corrected *P* < 0.05 were considered significantly enriched by the differential expressed genes. We used the software KOBAS (Mao et al., 2005) to test the statistical enrichment of the differential expression genes of the KEGG pathways.

### Quantitative real time-PCR validation for DEGs

To verify the gene expression data, we carried out quantitative real time-PCR (qRT-PCR) for 19 randomly selected DEGs, using 16S RNA (supplier, country) as the internal standard. We used the Primer Premier 5.0 software to design the oligonucleotides used to carry out the RT-qPCR. We used DNase I (supplier, country) to treat the total RNA from the blank sample and the ones containing formic acid (named Formic), furfural (named Furfural), and PHB (named PHB). The first-strand cDNAs were synthesized from 1,000 ng of RNA with the PrimeScript® RT reagents Kit (supplier, country) using gDNA Eraser (Perfect Real Time, DRR047A, TaKaRa, country) in a 20-μl reaction volume. Negative controls for the RT-qPCR were prepared without reverse transcriptase from the cDNA synthesis reactions. The RT-qPCR was carried out in an ABI StepOneTM Real-Time PCR System (Applied Biosystems, country) and the result was analyzed by the accompanying software. The amplification reactions were performed in a final reaction volume of 20 μl containing the SYBR® Premix Ex TaqTM II Kit (DRR820A, TaKaRa, country), using 2 μl of a 10-fold diluted cDNA solution described above as template, 10 μl of 2 × SYBR® Premix Ex TaqTM II (Tli RNaseH Plus, supplier, country), 0.4 μl of 50 × ROX Reference Dye (supplier, country), 6.0 μl of distilled water (dH2O) and 10 μM of each primer. The thermal cycling conditions were first 30 s at 95°C, followed by 40 cycles of 5 s at 95°C, and then 30 s at 60°C. Subsequently, the melting curve was recorded between 60 and 95°C. All the reactions were repeated three times in triplicate. We used the delta-delta-Ct method to calculate the relative expression levels for each gene and normalized the results to the internal control gene (Schmittgen and Livak, 2008).

## Results and discussion

### Sequencing and mapping of the *G. oxydans* NL71's transcriptome

The expression patterns of the genes in *G. oxydans* NL71 from the four samples were studied by sequencing the extracted RNA of the different samples. The procedure generated a total of 23,983,036, 25,394,500, 27,663,496, and 23,972,050 raw reads for the Blank, Formic, Furfural, and PHB samples, respectively.

An overview of the sequencing and assembly is outlined in Tables 1, 2. After removal of the adaptor sequences, duplication sequences, ambiguous reads, and low-quality reads, we generated 15,860,314, 16,147,218, 19,659,700, and 15,565,512 high-quality clean reads for the Blank, Formic, Furfural, and PHB samples, respectively. A proportion of 98% of the clean reads data presented Phred-like quality scores at the Q20 level (an error probability of 0.01) and 93% of the clean reads data exhibited Phred-like quality scores at the Q30 level (an error probability of 0.001). The number of clean reads that were aligned to the reference genome and annotated genes was determined by a detailed statistical analysis, which provided the general information for further transcriptome analysis. We mapped the generated reads to the total sequence of the *G. oxydans* NL71's genome (Miao et al., 2015) and obtained 89.98, 78.28, 91.02, and 90.14% of the total reads from the RNA-seq data for the Blank sample and the inhibitor pretreated samples with formic acid, furfural, and PHB, respectively. Complementarily, small proportions were multiply mapped to the genome. Unmapped or multiposition-matched reads were excluded for the subsequent analyses. The sequence data generated in this study were deposited at the NCBI in the Short Read Archive database under the accession number SRP060319.

**Table 1:** **Summary of the sequence assembly**.

**Sample name**	**Raw reads**	**Clean reads**	**Clean bases**	**Error rate (%)**	**Q20 (%)**	**Q30 (%)**	**GC content (%)**
Blank	23,983,036	15,860,314	1.59G	0.03	98.95	93.78	53.43
Formic	25,394,500	16,147,218	1.61G	0.03	98.67	94.18	47.07
Furfural	27,663,496	19,659,700	1.97G	0.03	99.05	94.50	53.31
PHB	23,972,050	15,565,512	1.56G	0.03	98.77	93.82	51.63

*The mentions “Blank,” “Formic,” “Furfural,” and “PHB” referred to the control sample and to the samples pretreated with formic acid, furfural, and 4-hydroxybenzaldehyde, respectively*.

**Table 2:** **Summary of RNA-sequencing data**.

**Sample name**	**Blank**	**Formic**	**Furfural**	**PHB**
Total reads	22,605,550	24,035,028	26,271,542	22,691,050
Total mapped	20,951,521 (92.68%)	19,296,934 (80.29%)	24,837,737 (94.54%)	20,989,972 (92.5%)
Multiple mapped	611,137 (2.7%)	482,773 (2.01%)	925,105 (3.52%)	536,272 (2.36%)
Uniquely mapped	20,340,383 (89.98%)	18,814,161 (78.28%)	23,912,632 (91.02%)	20,453,700 (90.14%)
Read-1	10,112,310 (44.73%)	9,294,293 (38.67%)	11,930,073 (45.41%)	10,161,333 (44.78%)
Read-2	10,228,074 (45.25%)	9,519,868 (39.61%)	11,982,559 (45.61%)	10,292,367 (45.36%)
Reads map to +	10,175,811 (45.01%)	9,414,519 (39.17%)	11,958,060 (45.52%)	10,231,590 (45.0%)
Reads map to –	10,164,573 (44.96%)	9,399,642 (39.11%)	11,954,572 (45.5%)	10,222,110 (45.05%)

*The mentions “Blank,” “Formic,” “Furfural,” and “PHB” refer to the control sample and to the samples pretreated with formic acid, furfural, and 4-hydroxybenzaldehyde, respectively*.

### Identification of differentially expressed genes between the four different samples

To measure the differences in gene expression levels based on the RNA-sequencing, we used the parameter RPKM (expected number of reads per kilobase of transcript sequence per millions base pairs sequenced) to quantify the transcripts expression. We assumed that the gene was expressed if the RPKM was superior to 1. We used all these uniquely mapped reads to calculate the RPKM-values of the genes. We considered that the genes with RPKMs in the range of 1–3 were expressed at a low level; genes with an RPKM-value between 3 and 15 were expressed at a middle level; and genes with an RPKM-value superior to 15 were expressed at a high level (Table 3). To minimize false positive and negative results, we concluded that a statistical analysis was reliable when it was applied to genes with an RPKM-value superior to 3 for at least one of the four samples.

**Table 3:** **Statistics of the genes at different expression-level intervals**.

**RPKM interval**	**Blank**	**Formic**	**Furfural**	**PHB**
0–1	75 (2.23%)	68 (2.03%)	65 (1.94%)	69 (2.05%)
1–3	40 (1.19%)	45 (1.34%)	16 (0.48%)	47 (1.40%)
3–15	696 (20.73%)	788 (23.47%)	379 (11.29%)	480 (14.29%)
15–60	1,282 (38.18%)	1,500 (44.67%)	1,562 (46.52%)	1,395 (41.54%)
>60	1,265 (37.67%)	957 (28.50%)	1,336 (39.79%)	1,367 (40.71%)

*The mentions “Blank,” “Formic,” “Furfural,” and “PHB” refer to the control sample and to the samples pretreated with formic acid, furfural, and 4-hydroxybenzaldehyde, respectively*.

**Figure 1:**
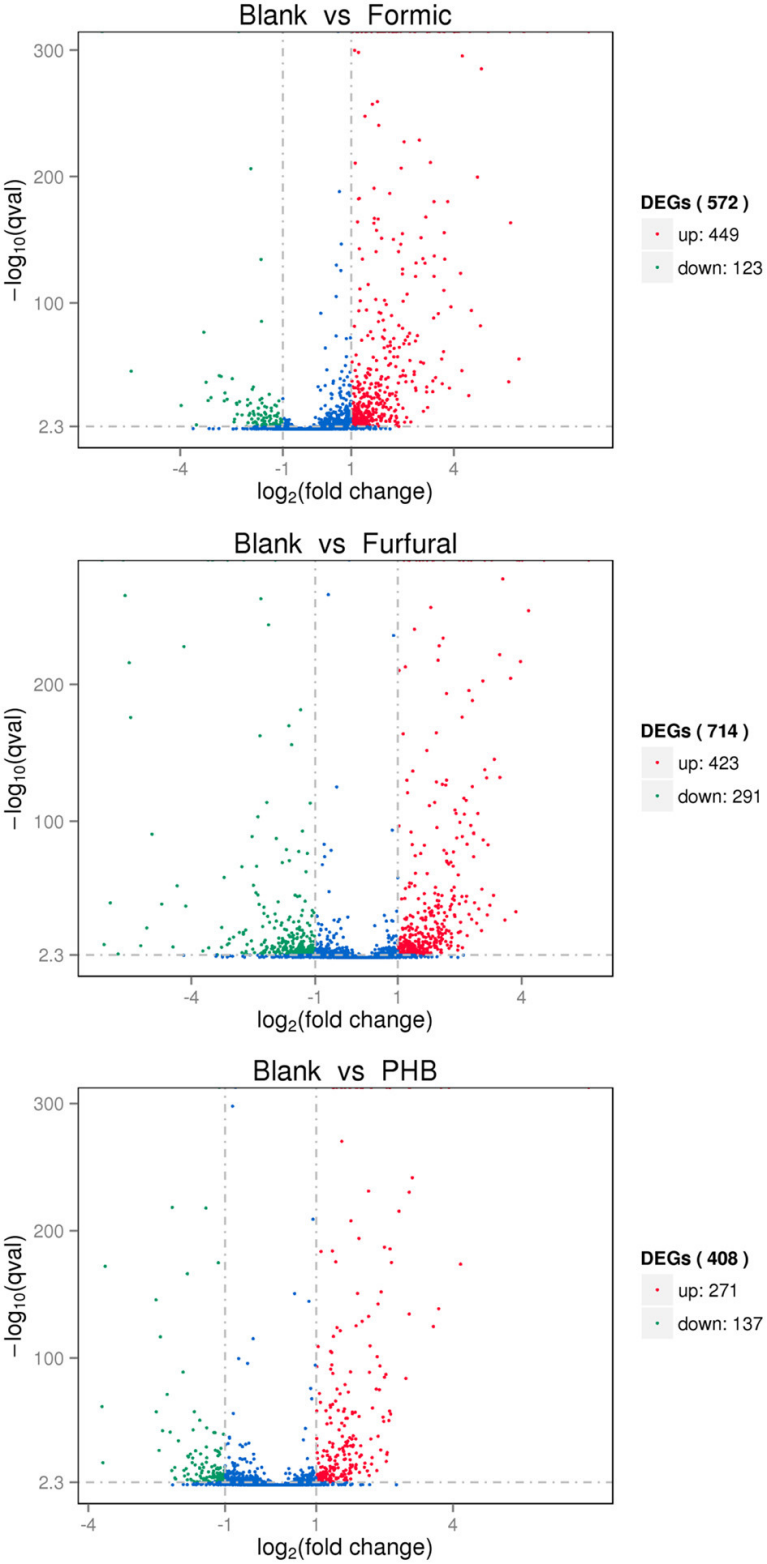
**Volcano plot displaying differential expressed genes between the blank sample and the inhibitor treated samples**. The *y*-axis corresponds to the mean expression value of the log_10_(*p*-value) and the *x*-axis displays the log_2_(Fold-change) value. The red dots represent the upregulated expressed genes, the green dots represent the downregulated expressed genes, and the blue dots represent the transcripts whose expression levels did not reach statistical significance between two samples.

The exploration of the biological mechanism of *G. oxydans* NL71 required the identification of the differentially expressed genes between the blank sample and the different inhibitor-treated samples. The differential gene expression profiles were examined by applying the trimmed mean of *M*-values (TMM) and DEGseq methods. Moreover, the corrected *P*-value was set to 0.005 and the log_2_ (Fold-change) to 1 as the threshold values to define the significantly differential expressions. As shown in the Figure 1, a total of 572 differentially expressed genes were detected by DEGseq between the Blank sample and the Formic sample. The expression level of 449 for 572 genes was upregulatedand the other 123 genes showed downregulated expression when treated with formic acid. When comparing the control sample with the samples pretreated with furfural and PHB, we obtained 714 DEGs (423 upregulated and 291 downregulated) and 408 DEGs (271 upregulated and 137 downregulated), respectively.

**Figure 2:**
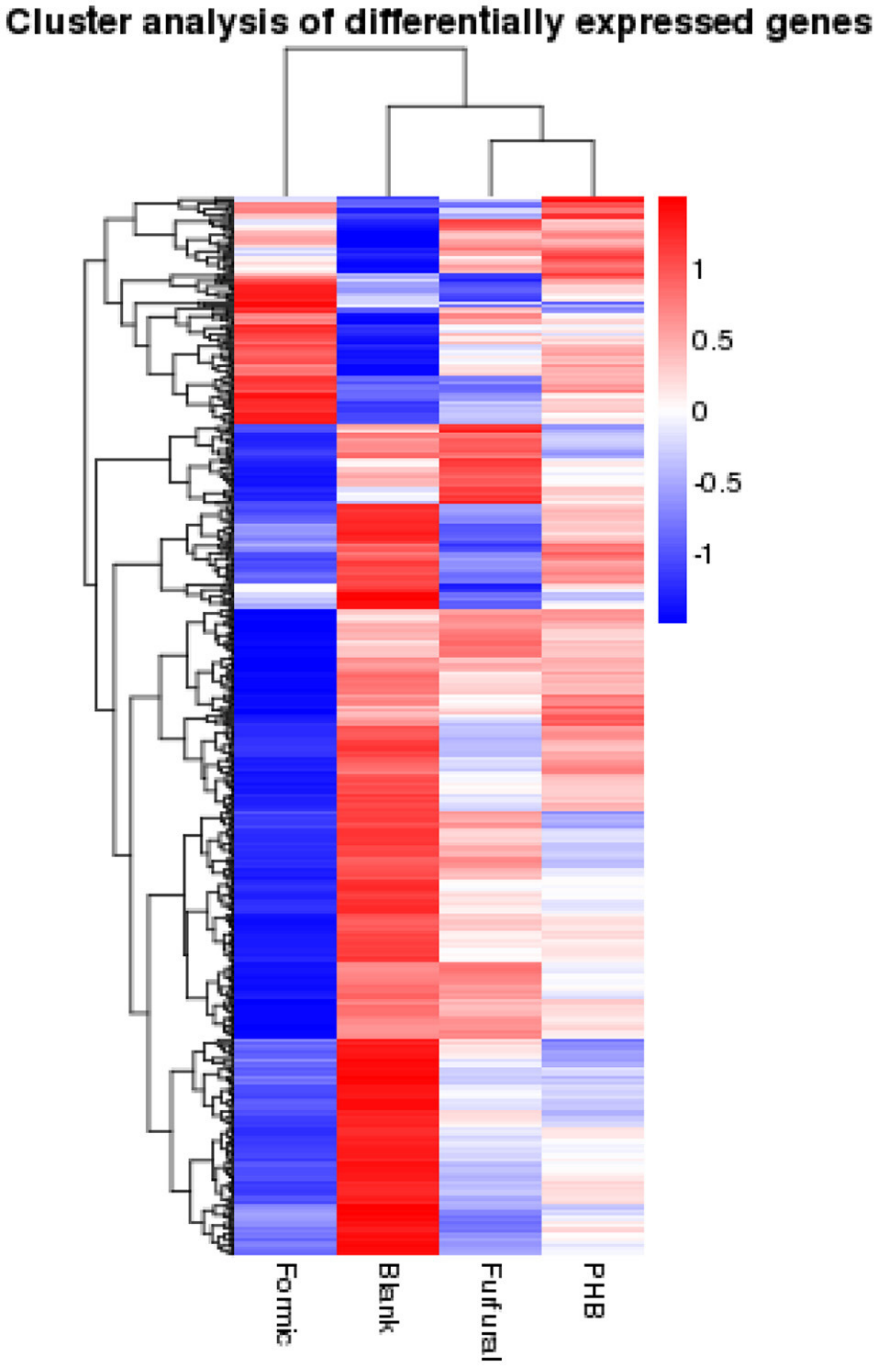
**Hierarchical clustering of the differentially expressed genes based on the log_**10**_RPKM-values**.

We then ordered the differentially expressed genes via a hierarchical clustering based on the four samples' log_10_ RPKM-values in order to extract the overall gene expression pattern as shown in the Figure 2. The low gene expression quantity was indicated by the blue bands and the high gene expression quantity was highlighted by the red ones (Figure 2).

**Figure 3:**
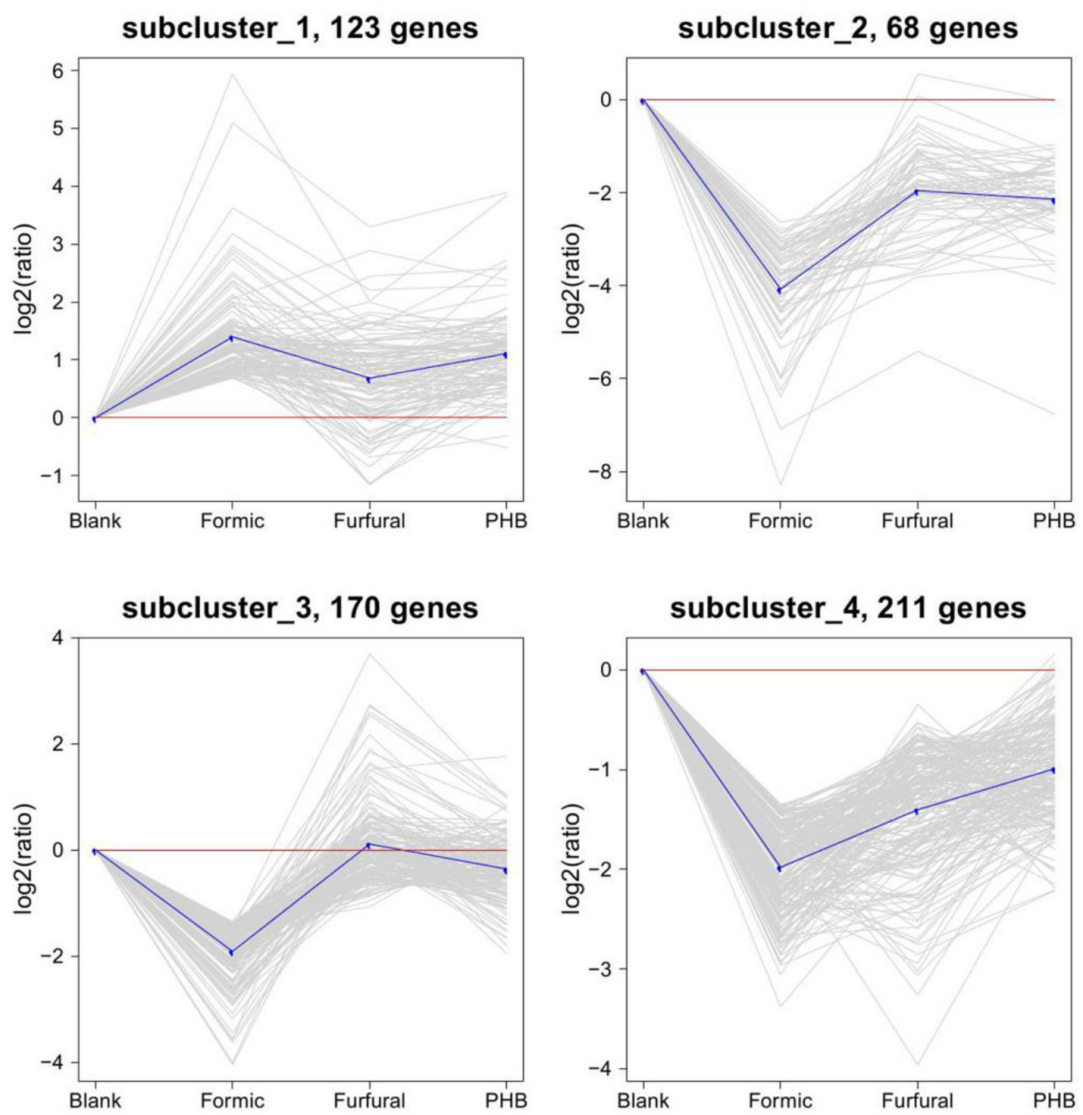
**Clustering of the differentially expressed genes**. The four major clusters obtained by the K-means algorithm. Expression ratios are expressed as log2.

The expression profiles of the DEGs were further determined by a cluster analysis based on the k-means method (Hartigan and Wong, 1979) to identify similar expression patterns from all of the DEGs across the set of stages and highlight the similarities and differences of the patterns obtained for the different DEGs. As shown on the expression patterns (clusters) of the differentially expressed genes identified in Figure 3, the most abundant group was the subcluster 4 containing 211 genes, whose expression was downregulated for the sample treated with formic acid and then upregulated for the ones treated with furfural and PHB. Subclusters 2 and 3 presented similar expressions as the subcluster 4 and consisted of 68 and 170 genes, respectively.

**Figure 4:**
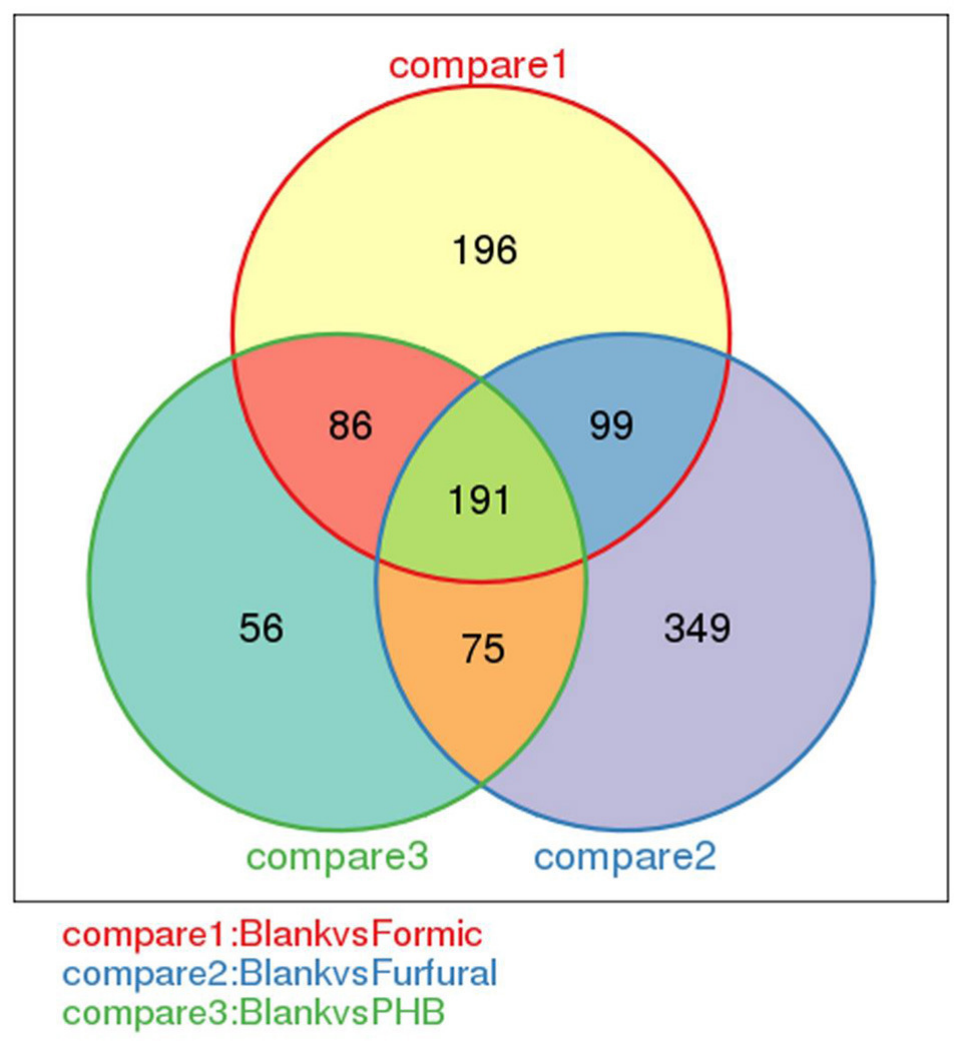
**Venn diagram showing the number of differentially expressed genes between every two samples**.

Comparison of the changes in gene expression between the control sample and the inhibitor-treated samples resulted in some similarities and considerable differences. The samples with and without inhibitor treatment showed transcriptome complexity. The Venn diagram shows the distribution of the expressed genes between the control sample and the inhibitor-treated samples (Figure 4). Hundreds of differentially expressed genes were detected between the control and the inhibitor-treated samples. Among these genes, 191 DEGs were expressed for all three comparison (Blank vs. Formic, Blank vs. Furfural, and Blank vs. PHB). When analyzing the control sample with the formic acid-treated sample against the control sample with the furfural-treated sample, a total of 290 genes were coexpressed. In the case of the analysis between the control sample compared with the furfural-treated sample against the control experiment compared with the PHB-treated sample, we found 277 and 266 coexpressed genes, respectively. These gene expression patterns illustrated the complexity of the inhibition mechanism during the D-xylonate production by *G. oxydans* NL71.

### Functional distribution of the differentially expressed genes

**Figure 5:**
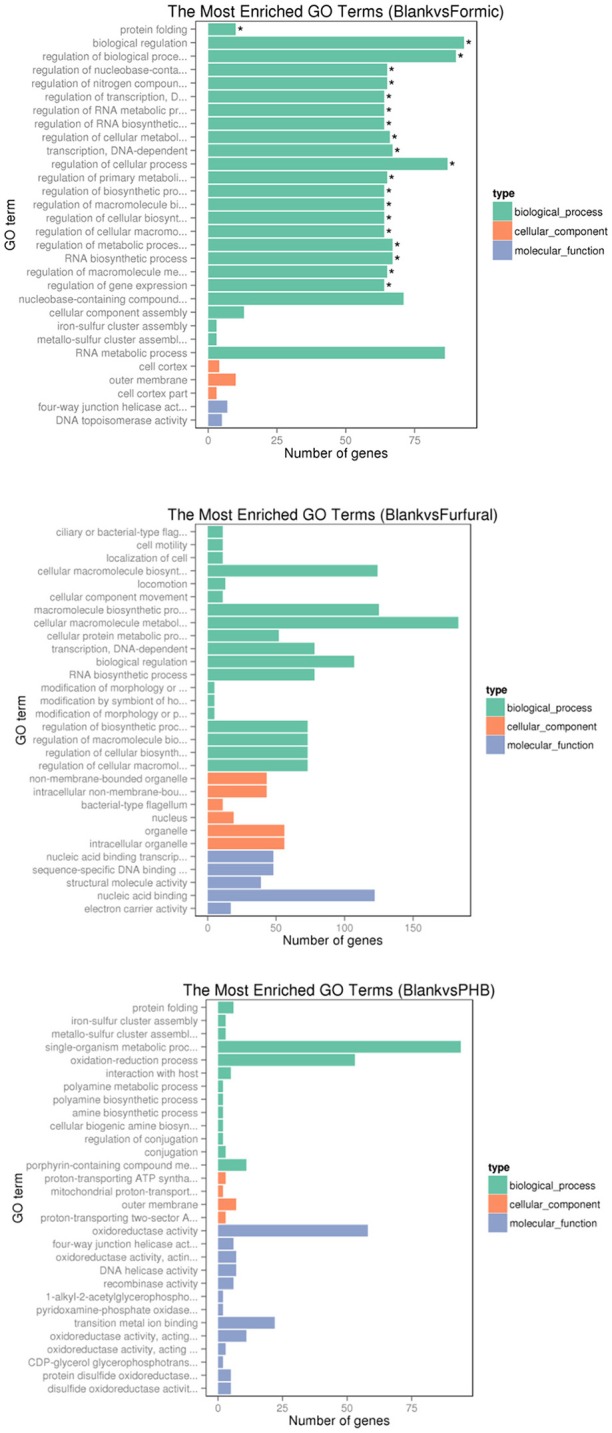
**Histogram of the gene ontology classification**. The results summarized the three main categories: biological process (BP), cellular component (CC), and molecular function (MF). The *X*-axis indicates the number of genes in a category. The left *Y*-axis indicates the second level term of gene ontology. ^*^The significantly enriched.

We used the GOseq R package to perform the gene ontology (GO) analysis based on the RNA-sequencing data. The GO classification for the differentially expressed genes was used to verify the annotations. As shown in the Figure 5, the top 30 most enriched GO terms were selected and clustered into three main categories, namely biological process, cellular component, and molecular function.

Comparison between the control sample and the formic acid-treated sample resultedin 25, 3, and 2 functional groups for biological process, cellular component, and molecular function categories, respectively. The top four functional categories included biological regulation (GO: 0065007), regulation of biological process (GO: 0050789), regulation of cellular process (GO: 0050794), and RNA metabolic process (GO: 0016070). When comparing the control experiment with the furfural-treated sample, the cellular macromolecule metabolic process (GO: 0044260) category registered 183 genes, whereas the cellular macromolecule biosynthetic process (GO: 0034645) contained 124 genes, and the macromolecule biosynthetic process (GO: 0009059) resulted in 125 genes. When comparing the control sample against the PHB treated sample, we noticed that the top three functional categories belonged to the single-organism metabolic process (GO: 0044710), oxidation-reduction process (GO: 0055114), and oxidoreductase activity (GO: 0016491).

The systematic analysis of the gene functions linked the genomic information to higher order function information that was active in the D-xylonic acid production process when the culture of *G. oxydans* NL71 was treated with the different inhibitors. Therefore, we mapped the detected genes on the reference metabolism pathways from the Kyoto Encyclopedia of Genes and Genomes (KEGG; http://www.genome.ad.jp/kegg/; Kanehisa et al., 2004) and compared them with the whole transcriptome background. When we analyzed the control experiment with the ones of formic acid, furfural, and PHB, the differentially expressed genes were assigned to 71, 54, and 59 KEGG pathways, respectively. These annotations provided a valuable resource for investigating specific processes, functions, and pathways involved in the D-xylonic acid production process when the culture of *G. oxydans* NL 71 was treated with an inhibitor.

### Effects of formic acid, furfural, and PHB on the global gene expression in *G. oxydans* NL71

There were many differentially expressed genes when we compared the ones of the control sample with the ones expressed in the samples treated with formic acid, furfural, and PHB (not listed). Genes were selected depending on the functional organization as described in the following paragraphs.

### Genes involved in the cellular respiration and energy metabolisms

Many of the genes whose expression was influenced by formic acid, furfural, and PHB were involved in the respiration and energy metabolisms. The respiratory chain was considered to be a key element to the oxidative metabolism in *G. oxydans* (Matsushita et al., 2004). Biochemical experiments and data from the genome-sequencing project enabled us to present a detailed model of the membrane-bound electron transport system (Prust et al., 2005). Several proteins directly fed electrons to the respiratory chain and many intracellular oxidoreductases showed decreased levels in the presence of the inhibitors. We also observed decreased expression levels of the genes coding for the membrane-bound PQQ-dependent alcohol dehydrogenase (NL71GM001165) and the membrane-bound lactate dehydrogenase (dld, NL71GM000996). Previous work reported the above-mentioned membrane-bound dehydrogenases were responsible for the rapid oxidation of ethanol and lactate. The opposite regulation of genes for these enzymes represented an adequate response to the electron flux into the respiratory chain (Adachi et al., 2007).

Notably, the genes encoding a proton-translocating nicotinamide dinucleotide transhydrogenase (pntB, NL71GM001999; pntB, NL71GM002001) belonged to the most strongly downregulated genes in the samples treated with the inhibitors. The transhydrogenases coupled the reversible hydride transfer between NADH + H^+^ and NAD^+^ to translocate protons across a membrane. In *G. oxydans*, the enzyme could fulfill two functions: (i) the oxidation of NADPH derived from intermediary metabolisms, and (ii) the transfer of protons across a membrane thereby contributing to the generation of the electrochemical proton gradient (Deppenmeier and Ehrenreich, 2009).

As described in previous reports, *G. oxydans* can express a variety of membrane-bound dehydrogenases that are relevant to the oxidation of sugars, alcohols, and polyols. However, we observed an alternative route, involving a second set of enzymes from the cytoplasm, which indicated that the uptake of the substrates into the cell was required. Some proteins involved in sugar and alcohol degradations showed an increased expression under the presence of inhibitors, such as the genes coding for the methylthioribulose-1-phosphate dehydratase (mtnB, NL71GM001007), the glycerol-3-phosphate dehydrogenase (glpA, glpD, NL71GM000162), the glycerol kinase (glpK, NL71GM000160), the D-ribulokinase (NL71GM002807), the L-xylulokinase (lyxK, NL7GM000175), and the glycerol-3-phosphate dehydrogenase (glpA, glpD, NL71GM000174). These proteins are NAD(P)^+^ dependent and can catalyze reversible reactions. The soluble NAD(P)^+^-dependent enzymes were considered to synthesize the biosynthetic precursors and were obviously involved in the maintenance of the cells during the stationary-growth phase. The substrates were oxidized and the resulting intermediates were phosphorylated and further metabolized via the pentose phosphate pathway.

Differentially expressed genes encoding enzymes that were involved in the Embden-Meyerhof-Parnas (EMP) and pentose-phosphate pathway (PPP), and the tricarboxylic-acid (TCA) cycles. Moreover, gluconeogenesis showed a higher level of expression in the presence of the inhibitors than untreated. The oxidative PPP is believed to be an important route for phosphorylative breakdown of sugars and polyols into carbon dioxide (Richhardt et al., 2013). The genes involved in the PPP were upregulated, like the one of the ribose 5-phosphate isomerase B (rpiB, NL71GM001290) and of the glucose-6-phosphate 1-dehydrogenase (zwf, NL71GM002174).

### Genes involved in the transport

We determined that *G. oxydans* possessed various transporters, symporters, and permeases for the absorption of substrates and ions such as metal ions, ammonium, phosphate, purines, amino acids, sulfate, pyrimidines, di- and oligopeptides, sugars, polyols, and sugar acids. In addition, we found some components of a phosphotransferase system. However, the phosphotransferase system in *G. oxydans* was considered to be an inactive transport system because of the lack of the EIIB and EIIC components (Prust et al., 2005).

The vast majority of the differentially expressed genes encoding enzymes involved in the transport of molecules were upregulated. Among these DEGs, we detected that the gene *feoA* and *feoB* (NL71GM000927 and NL71GM000928) encoding for ferrous iron transport proteins had increased expression levels. This observation could be related to an increased on the iron demand caused by the increased biosynthesis of the heme (Hanke et al., 2012). Several genes were involved in the protein secretion mentioned above such as the signal recognition particle protein (ffh, NL71GM002123), sec-independent protein translocase protein TatA (tatA, NL71GM001008), and preprotein translocase subunit SecA (secA, NL71GM000616).

### Genes involved in the biosynthesis of cofactors

Pyrroloquinoline quinone (PQQ) is an important prosthetic group involved in many membrane-bound dehydrogenases and is supposed to be crucial for the thriving of organisms. Therefore, an appropriate synthesis of the cofactor PQQ was essential for the correct function of quinoproteins. Proteins involved in the PQQ synthesis in *G. oxydans* were encoded by the *pqqABCDE* cluster (Felder et al., 2000; Prust et al., 2005). The PQQ backbone was constructed from glutamate and tyrosine speculatively derived from a small peptide encoded by the *pqqA* gene (Goosen et al., 1992). The gene encoding *pqqC* (pqqC, NL71GM001229) showed a decreased expression when the sample was treated with formic acid, which catalyzed the final formation step of PQQ (Magnusson et al., 2004). The protein PqqB could be involved in the transport of PQQ into the periplasm (Velterop et al., 1995) and the gene encoding *pqqB* (pqqB, NL71GM001228) was upregulated when the sample was treated with furfural. The genes encoding *pqqD* (pqqD, NL71GM001230) and *pqqE* (pqqE, NL71GM001231) were upregulated in the presence of the inhibitor. However, the functions of *pqqD* and *pqqE* remained unknown.

### Genes involved in the regulatory function and signal transduction

*G. oxydans* presented many two-component regulatory systems. They mediated the response to various environmental stimuli. The genome also coded for regulatory proteins. Many of them belonged to previously described regulator families such as LysR, AraC, MarR, TetR, AsnC, and MerR (Prust et al., 2005).

Several genes coding for transcriptional regulator families displayed an increased expression in the presence of an inhibitor, such as Fur (fur, NL71GM001482; zur, NL71GM001445), LysR (oxyR, NL71GM001607), DtxR (mntR, NL71GM000178), and DeoR (glpR, NL71GM000163). The protein functioned as a global regulator capable of sensing iron in iron homeostasis (Lee and Helmann, 2007).

Four genes involved in the signal transduction were upregulated. They belonged to two-component regulatory families such as NtrC (NL71GM002044), ChvI (NL71GM001436), and OmpR (NL71GM001422, NL71GM000691).

### Genes involved in the cell motility

*G. oxydans* appeared to have a chemosensory transducer protein system. The genome contained one complete set of chemotaxis genes that were organized in one gene cluster and three copies of genes encoding for methyl-accepting chemotaxis proteins (MCPs; Prust et al., 2005). On the basis of the experimental results, we observed that one gene encoding MCP (NL71GM000741) was upregulated in the presence of an inhibitor. However, only one gene encoding MCP (NL71GM001488) showed a decreased expression in the case of the sample treated with formic acid. The chemotactic response was completed by the signal transmission between the receptor complexes and the flagellar motor complexes (Bren and Eisenbach, 2000). As shown below, the chemotaxis families acted as messenger proteins, transducing the signal from the MCPs to the flagella, and were upregulated in the presence of an inhibitor except for CheB (NL71GM000736) and CheR (NL71GM000737).

Our experimental results confirmed the presence of peritrichous flagella as expected from the morphological features of *G. oxydans* (Asai et al., 1964). The genome of *G. oxydans* NL71 contained 22 predicted flagellar genes and most of the genes encoding for the structural proteins of the flagellum showed an increased expression level (Prust et al., 2005). These results suggested an increased in the motility of the cells, which could help the bacteria to change growth media rapidly.

### qRT-PCR validation of the RNA-Seq data

To identify the validity of the results obtained by RNA-Seq and to further evaluate the expression profiles of differentially expressed genes, the dependence of transcription changes of 19 selected genes from the four evaluated culture media (Blank, Formic, Furfural, PHB) was confirmed by qRT-PCR. The candidate genes for validation were chosen among representative genes involved in the pathway of the glycolysis, the TCA cycle, the PPP, the ABC transports, the bacterial secretion system, the bacterial chemotaxis, the electron transport, and the detoxification, which displayed increased and decreased transcription levels in response to the different inhibitors. As shown on Figure S1, the expression profiles of the 19 candidates were basically in accordance with the predictions obtained from the RNA-Seq results. Some genes showed different expression under the formic acid, furfural, and PHB conditions during the D-xylonate production. For example, the levels of NL71GM001713, NL71GM002001, NL71GM000337, and NL71GM000375 increased under the formic acid-treatment but decreased under the furfural and PHB treatments. Moreover, the selected genes displayed concordance and similar expression during the D-xylonate production when treated with the inhibitors.

## Conclusion

In conclusion, we investigated the whole genome transcriptome profiles of *G. oxydans* NL71 using RNA-sequencing to analyze and understand the transcriptional changes during the biooxidation of D-xylose into D-xylonate in critical culture media containing lignocellulosic-inhibitors. The RNA-sequencing data from a collection of *G. oxydans* NL71 generated new tools, which unveiled the genes responsible for the inhibitory effects associated to the bioconversion of D-xylose to D-xylonate by *G. oxydans* NL71. The RNA deep sequencing produced 24, 25, 28, and 24 million raw sequence-reads for the control sample and the ones treated with formic acid, furfural, and PHB, respectively. The digital gene expression profile screened 572, 714, and 408 DEGs when the control sample was compared to the samples treated with formic acid, furfural, and PHB, respectively. The results were assigned to three main categories following the gene ontology classification. A number of genes related to the cellular respiration and energy metabolisms (dld, pntB, mtnB, glpA, glpD, glpK, lyxK, rpiB, zwf), transport (feoA, feoB, ffh, tatA, secA), biosynthesis of cofactors (pqqB, pqqC, pqqD, pqqE), regulatory function and signal transduction (fur, zur, oxyR, mntR, glpR), and cell motility (cheB, cheR) could constitute the major reasons for explaining the cellular responses related to the inhibitory effects. The RNA-Seq data was validated by real-time quantitative PCR on 19 selected genes identified as up-regulated or down-regulated in response to the inhibitors during the D-xylonate production. This study greatly expanded the repertoire of the genes involved in the transcriptional response to different inhibitor treatments. The obtained results represented a valuable resource for the biological investigation of the inhibitory effect involved in *G. oxydans* as they facilitated the identification of the inhibitory effects on the D-xylonate production.

## Author contributions

YX designed the experiments and wrote the paper. YM carried out the experiments and wrote the paper. YS carried out the RT-PCR and wrote the paper.

### Conflict of interest statement

The authors declare that the research was conducted in the absence of any commercial or financial relationships that could be construed as a potential conflict of interest.

## Supplementary material

Figure S1 **Quantitative real time-PCR (qRT-PCR) of 19 genes randomly selected for Blank vs. Formic, Blank vs. Furfural, and Blank vs. PHB**. The relative expression level was assessed by qRT-PCR and shown by three replicates.
