# Supplementary figures and images for: Effects of Inhibitors on the Transcriptional Profiling of Gluconobater oxydans NL71 Genes after Biooxidation of Xylose into Xylonate
Source: Front Microbiol. 2017 Apr 25;8:716. doi: 10.3389/fmicb.2017.00716 (PMC5403930; doi:10.3389/fmicb.2017.00716)

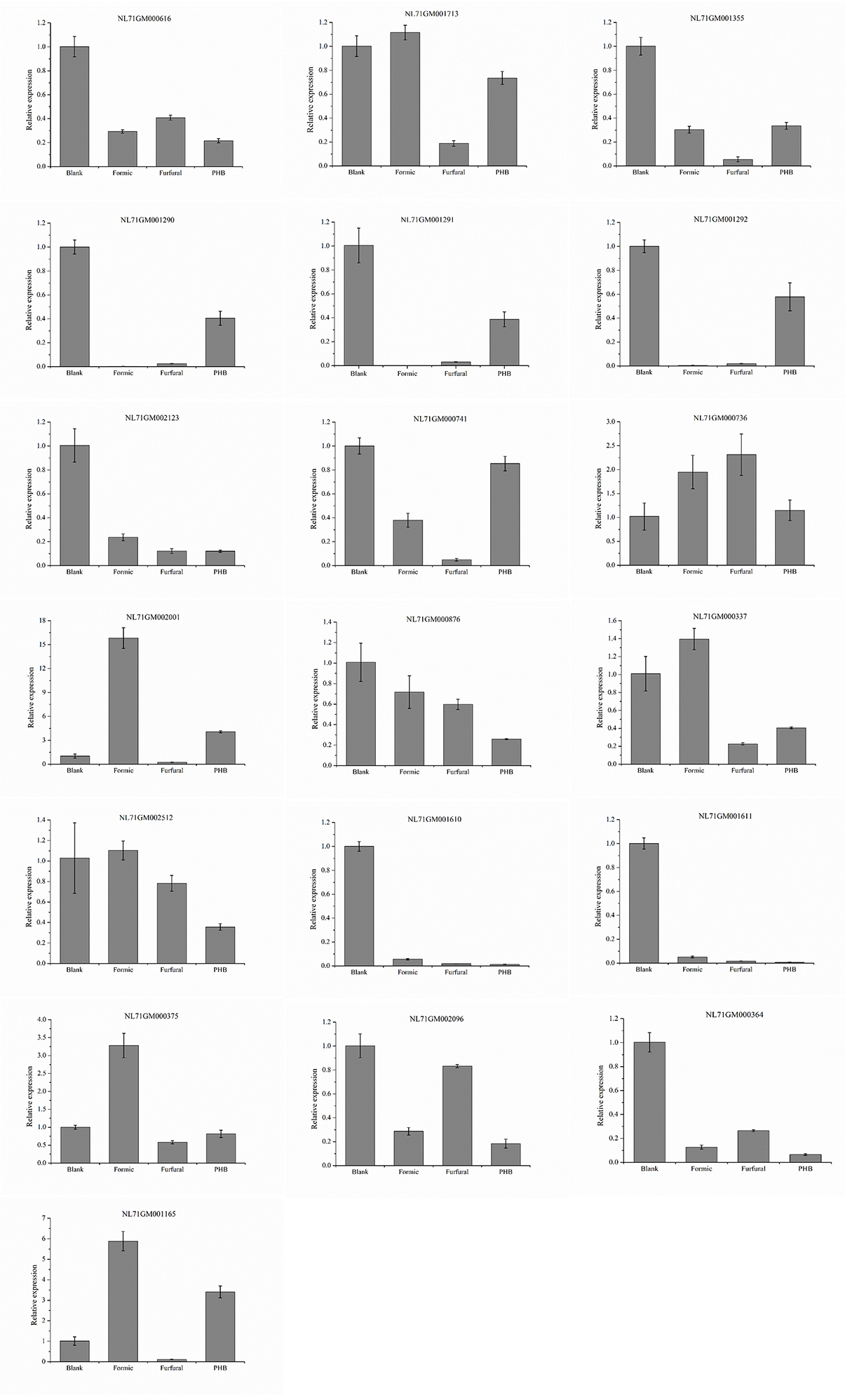

Supplement: Figure S1 — Quantitative real time-PCR (qRT-PCR) of 19 genes randomly selected for Blank vs. Formic, Blank vs. Furfural, and Blank vs. PHB. The relative expression level was assessed by qRT-PCR and shown by three replicates. [file Image1.TIFF]
